# Economic burden of childhood diarrhea in Burundi

**DOI:** 10.1186/s41256-021-00194-3

**Published:** 2021-04-12

**Authors:** Fulgence Niyibitegeka, Arthorn Riewpaiboon, Sitaporn Youngkong, Montarat Thavorncharoensap

**Affiliations:** 1 Master of Science Program in Social, Economic, and Administrative Pharmacy, Faculty of Pharmacy, Mahidol University, Bangkok, Thailand; 2 Division of Social and Administrative Pharmacy, Department of Pharmacy, Faculty of Pharmacy, Mahidol University, 447 Sri-Ayuthaya Road, Rajathevi, Bangkok, Thailand

## Abstract

**Background:**

In 2016, diarrhea killed around 7 children aged under 5 years per 1000 live births in Burundi. The objective of this study was to estimate the economic burden associated with diarrhea in Burundi and to examine factors affecting the cost to provide economic evidence useful for the policymaking about clinical management of diarrhea.

**Methods:**

The study was designed as a prospective cost-of-illness study using an incidence-based approach from the societal perspective. The study included patients aged under 5 years with acute non-bloody diarrhea who visited Buyenzi health center and Prince Regent Charles hospital from November to December 2019. Data were collected through interviews with patients’ caregivers and review of patients’ medical and financial records. Multiple linear regression was performed to identify factors affecting cost, and a cost model was used to generate predictions of various clinical and care management costs. All costs were converted into international dollars for the year 2019.

**Results:**

One hundred thirty-eight patients with an average age of 14.45 months were included in this study. Twenty-one percent of the total patients included were admitted. The average total cost per episode of diarrhea was Int$109.01. Outpatient visit and hospitalization costs per episode of diarrhea were Int$59.87 and Int$292, respectively. The costs were significantly affected by the health facility type, patient type, health insurance scheme, complications with dehydration, and duration of the episode before consultation. Our model indicates that the prevention of one case of dehydration results in savings of Int$16.81, accounting for approximately 11 times of the primary treatment cost of one case of diarrhea in the community-based management program for diarrhea in Burundi.

**Conclusion:**

Diarrhea is associated with a substantial economic burden to society. Evidence from this study provides useful information to support health interventions aimed at prevention of diarrhea and dehydration related to diarrhea in Burundi. Appropriate and timely care provided to patients with diarrhea in their communities and primary health centers can significantly reduce the economic burden of diarrhea. Implementing a health policy to provide inexpensive treatment to prevent dehydration can save significant amount of health expenditure.

## Introduction

Despite the remarkable achievements globally in the fight against diarrheal diseases in the last two decades, diarrhea remains as one of the major public health problems in the world [1, 2]. In 2016, it was estimated that diarrhea was responsible for approximately 446,000 deaths globally among children aged under 5 years [2]. However, despite the prevalence of diarrhea in all regions of the world, it has been reported that more deaths attributed to diarrhea occur in developing countries, especially in sub-Saharan Africa and south Asia [3]. For instance, in sub-Saharan Africa, diarrhea killed 290,724 children aged under 5 years or more than 65% of total global deaths [2]. Diverse factors have been reported, such as limited access to appropriate health care often due to financial issues, undernutrition, geographic inaccessibility, shortage of reliable access to safe water, and sanitation [4]. In Burundi, after malaria and pneumonia, diarrhea is the third leading cause of mortality among children under 5 years [5]. In 2016, it was estimated that the mortality rate due to diarrhea was 7 per 1,000 live births in Burundi [3].

In general, although diarrhea continues to kill people, it can be successfully prevented and treated [3, 6]. While the treatment of acute moderate diarrhea with oral rehydration salts has shown successful results, severe diarrhea often becomes complicated with dehydration and requires emergency care with intravenous fluids, or it can lead to death. However, in developing countries, it is not easy to get this type of emergency health care. Therefore, prevention and promptness of treatment are key strategies in the management of diarrhea to save the lives. World Health Organization (WHO) proposes the following prevention measures: use of rotavirus vaccine; access to safe drinking water; regular hand washing with soap; improved sanitation; exclusive breastfeeding for the first 6 months of life; good personal and food hygiene; and the promotion of health education on the transmission mode [6].

Burundi has made various efforts to improve public health; in 2006, with the purpose of improving access to health care, Burundi introduced a health care system to provide free services to all Burundian children aged under 5 years and to pregnant women seeking care at public health facilities [7]. However, the Burundian health system faces many challenges, such as a shortage of qualified health professionals, unequal distribution of existing staff between urban and rural areas, and a shortage of essential drugs [7]. In response to the WHO recommendation on the introduction of the rotavirus vaccine into all national immunization programs in 2009, Burundi introduced the rotavirus vaccine into the Expanded Program of Immunization (EPI) under Gavi support in December 2013 [8]. Furthermore, to ensure promptness of treatment, the Burundi government recently implemented a community-based management program for diarrhea in children aged under 5 years [9]. The program consists of treating all cases of moderate diarrhea occurring in children aged under 5 years with oral rehydration salts and zinc by community health workers in their communities [9]. However, the the program is still limited to some health districts.

Despite the introduction of rotavirus vaccine and other efforts made by the government of Burundi, the prevalence of diarrhea remains significantly high. This may be explained by the diversity of the viruses that causes diarrhea, seasonal variation, and climate change [10–12]. In 2016, according to the Burundi Demographic and Health Survey (DHS), 23% of all children aged under 5 years experienced at least one episode of diarrhea during the 2 weeks preceding the survey [13]. This suggests the necessity to increase the coverage of existing interventions and to implement new health interventions. However, extension or implementation of new interventions incurs new costs [14]. In the context of a limited budget, there is a need for evidence to guide policymaking and planning. Additionally, it has been demonstrated that costing studies, by indicating the economic burden of disease, can be a useful tool to raise awareness of the magnitude of the problem to decision-makers as well as to conduct cost-effectiveness analysis of potential health interventions in the prevention and treatment of diarrhea [15].

In Burundi, although the clinical burden of diarrhea is well known, the economic burden of diarrhea remains unknown. Therefore, this is a study in Burundi to provide economic evidence on the economic burden of diarrhea and the costs of clinical services and the management of diarrhea. The study aimed, firstly, to estimate total direct medical and non-medical costs and indirect costs related to the health care of diarrheal patients from the societal perspective; and secondly, to identify factors affecting costs in order to estimate the cost of clinical status as economic evidence to improve clinical management.

## Methods

### Study design and sites

This study was designed as a prospective cost-of-illness study using an incidence-based approach to calculate the total cost of a diarrheal episode. A societal perspective with a micro-costing approach was used to estimate the costs of diarrhea.

The study was carried out for a period of 2 months (November to December 2019) at two study sites located in Bujumbura, the economic capital city of Burundi, which were purposively selected: Prince Regent Charles Hospital (HPRC), which is a 600-bed tertiary care hospital, and Centre de Medecine Communautaire de Buyenzi (CMCB), which is a primary care health center.

### Study criteria

#### Inclusion criteria

Inclusion criteria were children aged under 5 years who visited the study health facilities, presented with acute non-bloody diarrhea as the first complaint, and whose caregivers consented to participate in the study.

#### Exclusion criteria

Exclusion criteria were children and their caregivers who were not able to communicate with the investigator in Kirundi, French or English, and children who presented with other acute diseases (e.g. Malaria) or very severe chronic diseases (e.g. HIV and malnutrition) for which diarrhea was assumed to be a related symptom or complication.

### Sample size estimation

To estimate the sample size, we used the formula as follows: (Z_α/2_σ/εμ)^2^, where Z_α/2_ is the critical value (1.96 for an α of 0.05), σ is the population standard deviation, μ is the population mean, and ε is the precision required [16]. Due to a lack of similar studies in Burundi, we referred to a study carried out in Libya [17], in which the mean cost was US$678.99, and the standard deviation was US$499.12. Therefore, for a precision of 15%, we estimated a minimum sample size of 93 patients. However, we recruited all the patients who consulted the study health facilities and met the study criteria within the study period.

### Cost valuation

Cost components included in this study were direct medical cost (i.e. medicines, investigation, and routine service costs), direct non-medical cost (i.e. transportation, meals, accommodation, extra diapers, and informal care costs), and indirect cost (i.e. productivity loss due to premature mortality).

For direct medical cost calculation, the quantities of resources used were multiplied by the unit cost of each medical service. Due to limitations in the calculation of actual unit cost of medical services, the unit cost of outpatient visit and bed-day was derived from WHO-CHOosing Interventions that are Cost-Effective (WHO-CHOICE) [18]. Unit costs for drugs and medical supplies were taken from the purchasing price of the study health facilities, while unit costs for laboratory tests were taken from private providers.

For direct non-medical cost calculation, costs were determined by the total expenses reported by the patient’s caregivers, obtained through interview. Regarding informal care cost estimation, the human capital approach was used to value the caregiver time loss [19]. The cost was estimated by multiplying the total number of days lost due to diarrhea by the daily Burundi per capita gross national income (GNI) [20].

Indirect costs due to premature death or mortality costs were calculated using the human capital approach [19]; the costs were a summation of the discounted value of per capita GNI during the period of working age. Forecasted per capita GNI was estimated based on an average rate of historical annual growth. A discount rate of 3% per year was used to convert future per capita GNI to present value [15].

The total direct medical cost was computed as the sum of the total drugs and medical supplies cost, total investigation cost, and total routine services cost at outpatient clinic and inpatient ward. The total direct non-medical cost was estimated by summing up the total cost of transportation, meals, accommodation, informal care, and other diarrhea-related expenses (e.g. extra diapers). The total cost per episode of diarrhea patient was computed as the sum of the total costs before, during, and after admission or visit. All costs collected in Burundian Francs (BIF) were converted to the international dollar (Int$) for the year 2019 using the purchasing power parity (PPP). The PPP conversion factor from the World Bank website was not yet available for the year 2019 at the time of analysis, thus, a proxy rate for the year 2018 was used (Int$1 = 651.052 BIF) [21]. Costs in the past were inflated using the Consumer Price Index (CPI) of Burundi [22].

### Data collection

The investigator introduced the study to the caregiver (parent or guardian) when the child was admitted to or visited the outpatient department for diarrhea. Then, we requested for consent to include the child in the study; if agreed, the caregiver was asked to sign a consent form. The data collection form was developed from the “WHO guidelines for economic burden of diarrheal disease with focus on assessing the costs of the rotavirus diarrhea” [23]. Data were collected by interviewing the patient’s caregivers and reviewing the patient’s medical and financial records. The collected data included patient demographics and clinical features (i.e. visit or admission information and resources used), health care-related expenses (i.e. treatment, food, transportation, accommodation, and extra diapers), and caregiver time loss.

### Data analysis

The SPSS version 21 was used for statistical analysis. Descriptive statistics were used to summarize all variables. Differences between study health facilities were compared using bivariate statistics. A *p*-value < 0.05 was used to indicate statistical significance. To explore the factors affecting the cost of diarrhea, we performed a multiple linear regression analysis using a stepwise method. Since the cost data were not normally distributed, a natural log transformation was used to meet the criteria of normal distribution. Model diagnostics were also applied to check quality of the cost functions [24]. Log cost was calculated by multiplying the unstandardized coefficient by the average value of each predictor. Estimation of the forecasted cost was done through retransformation of the log cost using anti-log (exponential) form and then adjusted by the smearing factor, which was calculated by averaging the exponential values of unstandardized residuals [24].

## Results

### Characteristics of included patients

A total of 138 patients were recruited for this study. Demographic and clinical characteristics of the patients are summarized in Table 1. Most of the patients were male (60.1%) and the average age for whole sample was 14.45 months. A majority of the patients (86%) were covered by the Free Health Care system. Between the different study sites, i.e. health center and hospital, there was no statistically significant difference regarding demographic characteristics of the patients. However, clinical characteristics of the patients differed significantly between the two health facilities (*p*-value < 0.05), except for the outcome on discharge and episode duration in terms of group. Twenty-eight patients (21.0%) were admitted with an average length of stay of 8.9 days. Sixty patients (43.48%) had complications of dehydration and all were treated at the hospital. Regarding the outcome on discharge, one patient died. The mean duration of symptoms before consultation at the health facilities was 2.99 days. The average duration of diarrheal episode was 7.02 days.

**Table 1:** Demographic and clinical characteristics of the patients

Characteristics	All (***n*** = 138)	Health center (***n*** = 44)	Hospital (***n*** = 94)	***P***-value*
**Demographic**				
Sex; n (%)				
Male	83 (60.14)	31 (70.45)	52 (55.32)	0.091**
Female	55 (39.85)	13 (29.54)	42 (44.68)	
Insurance during admission or visit; n (%)				
Free health care	119 (86.23)	44 (100)	75 (79.79)	0.016**
MFP	10 (7.25)	0 (0.0)	10 (10.64)	
Private insurance and personal payment	9 (6.52)	0 (0.0)	9(9.57)	
Age (months),				
Mean	14.45	13.42	14.94	0.712***
(SD, median)	(9.67,11.36)	(7.48,12.58)	(10.54,11.27)	
**Clinical**				
Patient type; n (%)				
Outpatient	109 (78.99)	44 (100)	65 (69.15)	< 0.001**
Inpatient	29 (21.01)	0 (0.0)	29 (30.85)	
Complication with dehydration; n (%)				
Without dehydration	78 (56.52)	44 (100)	34 (36.17)	< 0.001**
With dehydration	60 (43.48)	0 (0.0)	60 (63.83)	
Discharge outcome; n (%)				
Alive	137 (99.28)	44 (100)	93 (98.94)	1.000****
Dead	1 (0.72)	0 (0.0)	1 (1.06)	
Duration group before visit or admission; n (%)				
< 2 days	73 (52.90)	27(61.36)	46 (48.94)	0.173**
> 2 days	65 (47.10)	17 (38.64)	48 (51.06)	
Duration before visit or admission; day				
Mean	2.99	2.30	3.19	0.030***
(SD, Median)	(2.76, 2.00)	(1.96, 2.00)	(3.02, 3.00)	
Episode duration; day				
Mean	7.02	6.07	7.47	0.004***
(SD, Median)	(4.97),6	(5.27),4.50	(4.79),6.00	

*MFP* “Mutuelle de la Fonction Publique”, a health insurance scheme for civil servants, *SD* standard deviation

^*^To compare between health center (CMC Buyenzi) and hospital (Prince Regent Charles), a *p*-value < 0.05 was used as statistical significance

^**^ Chi-square

^***^ Mann-Whitney U test

^****^ Fisher’s Exact test

### Health care services and resources utilization

Table 2 shows major health care services and resource utilization in the two study health facilities. Overall, antibiotics were prescribed for 61.59% of cases and were more likely to be prescribed at the health center (97.73%) compared to the hospital (44.67%) (*p* < 0.001). The most commonly used antibiotics were co-trimoxazole oral suspension (26.81%) followed by ampicillin and gentamicin injections at a rate of 19.57 and 10.14%, respectively. The prescription patterns between the health center and hospital were significantly different (*p* < 0.001). Laboratory tests were requested for 67.39% of total cases, and the most common test was full blood count (43.48%) followed by C-reactive protein (40.58%) and thick blood smear for malaria microscopy (40.58%). Full blood count and C-reactive protein were exclusively requested at the hospital, while malaria rapid test was performed only at the health center. There were significant differences for all laboratory tests between the health center and hospital (*p* < 0.001). Intravenous fluids were given to 43.48% of patients, all at the hospital, while oral rehydration salts were given to 26.81% of patients. Most of the other medicines prescribed were different compounds of zinc (29.71%) followed by paracetamol syrup (20.29%).

**Table 2:** Major health care services and resource utilization

Characteristics	All (***n*** = 138)	Health center (***n*** = 44)	Hospital (***n*** = 94)	***P***-value*
Antibiotics; n (%)	85(61.59)	43(97.73)	42(44.68)	< 0.001**
Co-trimoxazole suspension 40 mg/200 mg per 5 ml	37(26.81)	36(81.82)	1(1.06)	< 0.001**
Ampicillin injection 1 g	27(19.57)	0(0.0)	27(28.72)	< 0.001**
Gentamicin injection 1 vial 80 mg	14(10.14)	0(0.0)	14(14.89)	0.005**
Metronidazole syrup 125 mg 100 ml	14(10.14)	10(22.73)	4(4.26)	0.002****
Amoxicillin syrup 125 mg/5 ml	10(7.25)	4(9.09)	6(6.38)	0.726****
Cefotaxime injection 1 g IM/IV	7(5.07)	0(0.0)	7(7.45)	0.097****
Metronidazole injection .500 mg/100 ml	7(5.07)	0(0.0)	7(7.45)	0.097****
Laboratory tests (%)	93(67.39)	30(68.18)	63(67.21)	0.892**
Full blood count	60(43.48)	0(0.0)	60(63.83)	< 0.001**
C-reactive protein (CRP)	56(40.58)	0(0.0)	56(59.57)	< 0.001**
Thick blood smear	56(40.58)	5(11.36)	51(54.26)	< 0.001**
Stool microscopy	33(23.91)	24(54.55)	9(9.57)	< 0.001**
Malaria rapid test	15(10.87)	15(34.09)	0(0.0)	< 0.001**
Ringer lactate 500 ml	60(43.48)	0(0.0)	60(63.83)	< 0.001**
Paracetamol syrup 120 mg/5 ml	28(20.29)	27(61.36)	1(1.06)	< 0.001**
ORS	37(26.81)	33(75.00)	4(4.26)	< 0.001**
Zinc sulfate 20 mg tablet	41(29.71)	32(72.73)	9(9.57)	< 0.001**
Length of stay (day) ^a^; mean (SD), median	8.90 (3.6),9.0	n/a	8.90 (3.6),9.0	n/a
Treatment before visit or admission; n (%)	51(36.96)	6 (13.64)	45 (47.87)	< 0.001**
Treatment after admission/visit; n (%)	7 (5.07)	0 (0.0)	7 (7.45)	0.097****
Caregiver’s time loss (day); mean (SD), median	7.45 (8.91),4	2.72 (1.89),2	9.66 (9.98),5	< 0.001***

*n/a* not applicable, *SD* standard deviation, *ORS* Oral rehydration salts

^*^To compare between health center (CMC Buyenzi) and hospital (Prince Regent Charles), a *p*-value < 0.05 was used as statistical significance

^**^ Chi-square

^***^ Mann-Whitney U test

^****^ Fisher’s Exact test

^a^for inpatients

Overall, 36.96% of patients sought treatment or care before visit or admission to the study health facilities. However, when compared to the health center, patients who consulted at the hospital were more likely to have received care before (*p* < 0.001). Additional care from other health facilities after receiving treatment at the study health facilities was sought by only 5.07% of total patients. The average time loss of caregivers was 7.45 days. Caregiver time loss for the hospital patients was higher than that of the health center patients (*p* < 0.001).

The unit cost of commonly used drugs, medical consumables, laboratory tests, and services are presented in Table 3.

**Table 3:** Unit cost of major service and resource utilization (value in 2019)

Item name	Unit	Unit cost (Int$)	
		Health center	Hospital
**Drugs**			
syrup 125 mg/5 ml	100 ml bottle	1.53	2.02
Ampicillin injection 1 g	1 g-vial	n/a	0.82
Cefotaxime injection 1 g IM/IV	1 g-vial	n/a	1.30
Gentamicin injection 1 vial 80 mg	80 mg-vial	n/a	0.34
Metronidazole syrup 125 mg 100 ml	100 ml bottle	1.44	1.37
Metronidazole injection 500 mg/100 ml	100 ml bottle	n/a	1.21
Paracetamol syrup 120 mg/5 ml	100 ml bottle	1.42	1.13
Co-Trimoxazole 40 mg/200 mg per 5 ml	50 ml bottle	1.45	1.45
Ringer lactate solution	500 ml	n/a	1.93
ORS	1 packet	0.40	0.40
Zinc sulfate 20 mg	1 tablet	0.06	0.06
**Medical supplies**			
Catheter court IV UU 24 g	1 piece	n/a	0.44
Examination gloves	1 pair	0.65	0.65
Syringe a UU 5 ml	1 piece	n/a	0.14
**Laboratory tests**			
Stool microscopy	1 test	5.38	5.38
Thick blood smear	1 test	3.07	3.07
Full blood count	1 test	15.36	15.36
C-reactive protein (CRP)	1 test	15.36	15.36
Malaria Rapid test	1 test	7.68	7.68
**Routine OPD service**	1 visit	1.35	1.98
**Routine IPD service**	1 bed day	n/a	4.44
**Informal care**	1 day loss	2.04	2.04

*n/a* not available

### Description of cost

Table 4 summarizes different types of cost per diarrheal episode by health facility type. The average direct medical cost per diarrheal episode was Int$77.24. Before visit or admission, 36.96% of patients sought medical care, and the average cost was Int$5.73. During admission, the average medical cost was Int$71.42. Drugs and medical supplies formed a majority of the cost during admission with an average amount of Int$39.92, followed by investigation cost estimated to be Int$22.07 and routine service cost of Int$9.43. After visit or discharge, 5.07% of all patients sought medical care with an average medical cost of Int$0.09. Comparing the two health facilities, direct medical cost per each type and component was significantly higher for the hospital, except for the cost after visit or discharge.

**Table 4:** Cost per episode (Int$ in 2019) by health facility type and cost components

Type of cost	All (***n*** = 137)			Health center (***n*** = 44)			Hospital (***n*** = 94)			***P***-value*
	Mean	SD	Median	Mean	SD	Median	Mean	SD	Median	
Direct medical cost	77.24	94.92	41.64	15.35	8.80	15.06	106.52	102.88	71.67	< 0.001
Before visit/admission	5.73	12.32	0.00	1.83	5.35	0.00	7.57	14.15	0.00	< 0.001
Visit/admission	71.42	92.36	39.99	13.53	7.13	12.23	98.82	101.12	66.24	< 0.001
Drugs and medical supplies	39.92	63.43	13.39	5.40	2.36	4.82	56.26	71.46	33.87	< 0.001
Investigation cost	22.07	24.19	13.06	6.63	5.39	5.38	29.38	26.14	35.33	< 0.001
Routine services	9.43	15.93	1.98	1.50	0.43	1.35	13.18	18.19	1.98	< 0.001
After visit/admission	0.09	0.83	0.00	0.00	0.00	0.00	0.13	1.00	0.00	0.332
Direct non-medical cost	31.77	36.82	18.57	9.32	6.64	8.59	42.39	40.36	30.06	< 0.001
Transportation^a^	9.36	14.51	4.61	1.60	2.22	1.08	13.03	16.33	7.53	< 0.001
Meal^a^	3.58	6.79	0.92	0.80	2.41	0.00	4.90	7.74	2.30	< 0.001
Accommodation^a^	0.00	0.00	0.00	0.00	0.00	0.00	0.00	0.00	0.00	1.000
Informal care^a^	15.90	19.17	8.59	5.83	4.05	4.29	20.66	21.54	10.74	< 0.001
Others^a^	2.93	9.42	0.00	1.09	4.08	0.00	3.80	11.00	0.00	0.205
Total cost per episode	109.01	123.35	59.59	24.67	12.70	23.82	148.91	131.93	98.64	< 0.001

*SD* standard deviation

*to compare between health center and the hospital with Mann-Whitney test, a *p*-value < 0.05 was used as statistical significance

^a^ include the cost before, during and after visit or admission

The average direct non-medical cost was Int$31.77 per diarrheal episode. Informal care was the largest component with a cost estimated to be Int$15.90, followed by transportation and meal expenses with an average cost of Int$9.36 and Int$3.58, respectively. When comparing between health center and hospital, there was a statistically significant difference in terms of total direct medical cost, transportation, and meal-related expenses, as well as informal care cost for the whole diarrheal episode. No significant statistical difference was observed for other diarrhea-related expenses when comparing the two health facilities, and the overall average cost was Int$2.93. There was no report on accommodation expenses related to the illness for all patients included in this study.

The cost per type of clinical service and health facility is presented in Table 5. The average cost per episode of outpatient visit and inpatient admission was Int$59.87 and Int$292, respectively. The cost of outpatient visit at the hospital and health center (Int$84.07 versus Int$24.67) was significantly different.

**Table 5:** Distribution of cost per episode (Int$ in 2019) by type of patients and health facility

Patient type	All (***n*** = 137)				Health center (***n*** = 44)				Hospital (***n*** = 93)				***p***-value*
	n	Mean	SD	Median	n	Mean	SD	Median	n	Mean	SD	Median	
**Outpatient**													
DMC	108	39.71	37.06	24.77	44	15.35	8.80	15.06	64	56.46	39.76	50.49	< 0.001
DnMC	108	20.16	22.14	13.89	44	9.32	6.64	8.59	64	27.61	25.77	19.41	< 0.001
Total cost	108	59.87	51.00	43.52	44	24.67	12.70	23.82	64	84.07	53.37	70.61	< 0.001
**Inpatient**													
DMC	29	217.01	113.34	182.94	00	n/a	n/a	n/a	29	217.01	113.34	182.94	n/a
DnMC	29	74.99	47.51	59.54	00	n/a	n/a	n/a	29	74.99	47.51	59.54	n/a
Total cost	29	292.00	141.29	239.02	00	n/a	n/a	n/a	29	292.00	141.29	239.02	n/a

*n/a* not applicable (no inpatient care at health center), *DMC* Direct medical cost, *DnMC* Direct non-medical cost, *SD* standard deviation

*To compare between outpatient health center and outpatient hospital employing Mann-Whitney test, a *p*-value < 0.05 was used as statistical significance

For one fatal case, the indirect cost that included productivity loss due to premature death was Int$31,963.20. The total cost of illness, including direct costs incurred before death and indirect cost, was estimated to be Int$32,000.53.

### Estimates of annual economic burden of diarrhea in Burundi

As this study was conducted at a primary health center and a tertiary hospital, to estimate the cost of diarrhea in secondary care (i.e. district hospital), our estimated cost of diarrhea for a tertiary hospital was adjusted by the ratio of the unit cost at secondary hospital (Int$2.01) and tertiary hospital (Int$2.1) from WHO-CHOICE estimates [18]. Therefore, the cost of outpatient visit at district hospital was estimated to Int$84.07 × 2.01/2.10 = Int$80.47. The admission cost at district hospital was estimated to Int$292 × 2.01/2.1 = Int$279.49. According to the DHS [13], of the total patients with moderate diarrhea, the proportion of utilization of outpatient services was 92.87, 5.02, and 2.11% for health center, district hospital, and tertiary hospital, respectively. The nationwide cost of outpatient visit was therefore estimated to Int$24.67 × 0.9287+ Int$80.47 × 0.0502+ Int$84.07 × 0.0211 = Int$28.72. The proportions of utilization of inpatient admissions were 70.39, and 29.61% for district and tertiary hospital, respectively. Therefore, the average nationwide cost for inpatient cost was estimated to Int$279.49 × 0.7039 + Int$292 × 0.2961 = Int$283.19. Regarding mild diarrhea, which does not require medical attention, medication cost was assumed to be zero, according to the DHS. However, a loss of income (Int$2.14) due to absence from work was assumed at half of the average duration patients waited for a consultation at the study health facilities.

From the DHS [13], 41% of diarrheal episodes were mild and, therefore, did not require consultation for medical care. The remaining 59% required care from health facilities. According to this study, of the total patients included, 21% were admitted and 79% were treated within the outpatient visit. Therefore, we estimated the proportions of diarrheal cases requiring hospitalization and those managed within the outpatient visit at 21 × 59/100 = 12%, and 79 × 59/100 = 47%, respectively.

According to the DHS [13], the incidence of diarrhea was 3.9 episodes per person-year. With a population of children under 5 years estimated to be 2,230,568 in 2019 [25], the total number of diarrheal episodes was estimated to be 8,681,336 and composed of 3,559,348 mild cases managed at home; 4,046,371 outpatient visits; and 1,075,618 admissions. The mortality rate from diarrheal diseases in Burundi was 7 per 1,000 live births according to a UNICEF report [3], resulting in 3,327 deaths in 2019. The annual economic burden of diarrhea was, therefore, estimated to Int$534,915,134 (3,559,348 mild cases x Int$2.14 + 4,046,371 outpatient cases x Int$28.24 + 1,075,618 admitted cases x Int$259.72 + 3327 fatal cases x Int$32,000.53).

### Analysis of factors affecting cost

As shown in Table 6, six variables significantly positively affected the total cost-of-illness: health facility type, patient type, duration of the episode before consultation, antibiotics use, health insurance scheme (MFP), and complications with dehydration. Adjusted *R*^*2*^ was 0.773. The variables excluded from the final model were age and private insurance.

**Table 6:** Multiple linear regression analysis of the cost

Variables	β	SE	***P***-Value
Constant health facility type (ref: health center)	8.991	0161	< 0.001
Hospital	0.907	0.16	< 0.001
Patient type (ref: outpatient)			
Inpatient	1.28	0.17	< 0.001
Antibiotics use (ref: **N**o use)			
Use	0.451	0.138	< 0.001
Episode before consultation (ref: episode ≤2 days)			
Episode> 2 days	0.285	0.092	0.002
Insurance scheme (ref: free health care scheme)			
MFP	0.589	0.182	0.002
Complication with diarrhea (ref: no dehydration)			
Dehydration	0.275	0.127	0.032

Dependent variable = Natural logarithm of total cost of illness of diarrhea

Adjusted *R*^2^ = 0.773 (*R*^2^ = 0.778)

*MFP* “Mutuelle de la Fonction Publique”, a health insurance scheme for civil servants, *Ref* reference

A *p*-value < 0.05 was used as statistical significance

For analysis of assumptions and model diagnostics [24], no funnel shape was observed for the scatter plot of residuals confronted to predicted values and all predictors, confirming homoscedasticity. The test of independence of residuals was shown by the Durbin-Watson value of 2.034, which falls in the acceptable range (1.5–2.5). The condition index was 10.47, and therefore, the condition for no multicollinearity that requires the condition index to be less than 30 was met. The average Cook’s distance was 0.008 and the range was 0.000–0.07. Therefore, the condition of less than 1, indicating no influential observation, was observed.

In clinical practice, oral rehydration salts (ORS) are recommended for diarrhea to prevent dehydration. To provide economic evidence of the significance of ORS, the costs of patients with dehydration and without dehydration were estimated. For other predictor variables, average values were assumed constant. As a result, the costs of patients with dehydration and without dehydration were Int$69.92 and Int$53.11, respectively. The resulting difference (Int$16.81) was savings if dehydration was prevented. In the community-based management of diarrhea, a program being implemented in some health districts, a diarrheal patient aged 2 to 11 months is treated using 1 packet of ORS per day for 3 days and 1 half-tablet of 20 mg of zinc sulfate per day for 10 days, while those aged 1 to 5 years receives 1 packet of ORS per day for 3 days and 1 tablet of 20 mg of zinc sulfate per day for 10 days. Assuming the equal distribution of children under the age of five (children aged 2 to 11 months = 20%, children aged 1 to 5 years = 80%), the average primary drug cost per one case of diarrhea is estimated to be Int$1.48 (ORS Int$0.40 per packet × 3 packets) + [(zinc sulfate Int$0.06 per tablet × 5 tablets) × 20% of children aged 2 to 11 months) + (zinc sulfate Int$0.06 per tablet × 10 tablets) × 80% of children aged 1 to 5 years)] (prices of drugs refer to Table 3). From the total annual number of diarrheal episodes estimated to be 8,681,336, we estimated that 5,121,989 episodes (8,681,336 × 59%) would require medical care, according to the DHS [13]. With the proportion of patients with dehydration estimated to be 43.48% of the total diarrheal patients who consult for medical care, we estimated the annual number of cases of dehydration to be 2,227,041 cases (5,121,989 × 43.48%). Assuming a coverage and an effectiveness of 100% for dehydration prevention, an ORS program would save an amount of Int$37,436,559.21 (Int$16.81 × 2,227,041 cases) at the national level. In terms of public health management, the cost of the dehydration prevention program should be less than this amount of savings generated by the program outcome.

To extend the significance of our model and its application to clinical management, we also compared two scenarios. We considered late treatment at the inpatient department of a hospital after 2 days of diarrheal onset, with dehydration and antibiotics use, as the worst-case scenario. We considered early treatment at the outpatient department of a health center within 2 days of diarrheal onset, without dehydration and antibiotics use, as the best-case scenario. Our model predicted the costs resulting from the two scenarios to be Int$354.81 and Int$14.49 for the worst- and the best-case, respectively. The difference (i.e. Int$340.32) can be assumed to be savings resulting from switching from late treatment at hospital to early treatment at health center for one case. This finding shows significance in economic benefit of providing adequate and accessible primary care.

## Discussion

This is a study to provide evidence on the economic burden of diarrhea in Burundi. Overall, our results indicate that diarrhea is associated with a substantial burden on both the families and the country. For instance, we found that the average cost per episode of diarrhea was Int$109.01, which accounts for 14.5% of the GNI per capita [20]. Our study also estimated the annual economic burden of diarrhea at the national level; we found that, each year, diarrhea imposes a burden of Int$534,915,134, accounting for 6.4% of Burundi’s GNI in 2018 [26]. However, our estimation did not take into account costs in the private sector that were previously revealed to be associated with higher cost when compared to the public sector [27–29]. Therefore, our findings could be underestimated. Contrary to some previous studies, which found that direct non-medical costs, including informal care cost, were the largest cost component [30, 31], we found that direct medical costs were the largest component of the total cost of diarrhea. Direct medical cost accounts for 70.86% of the total cost, and the largest driver of direct medical cost was drugs and medical supplies cost (59.2%). This could be explained by low socioeconomic conditions of our participants, resulting in low direct non-medical cost. Burundi is one of the countries with the lowest GDP per capita; thus, most of the caregivers might opt to walk to receive treatment, eat at home, and use washable cloth instead of single-use diapers. In addition, most of the caregivers were not employed and none reported their accommodation cost. On the other hand, although not explored in our study, since most of the medicines used in Burundi are imported, the price of medicines might be exorbitant.

Despite the variability of the methodology applied, our findings were somewhat similar to previous studies identified in developing countries [17, 27, 29, 31–42]. To allow for comparison of our estimated costs with results of other previous studies, we converted all results into international dollars in 2018, using the specific inflation rate and purchasing power parity rate reported for each country [21, 43]. Our estimated cost per diarrheal episode (Int$109.01) falls within the range of findings of previous studies. Fom the societal perspective, the average cost per episode of diarrhea was estimated to be Int$9.03, Int$17.56, and Int$9.62 in Gambia, Kenya, and Mali, respectively [35]. In Bangladesh, the average cost per diarrheal episode was Int$126.95 [39]. In Vietnam, from the societal perspective, one study [29] estimated the cost per episode to be Int$213.08 and another study [32] found the cost to be Int$528.96 per episode. However, direct comparisons between these studies should be performed carefully since design, sites, scope of resource and service utilization, and socioeconomic characteristics of each study may vary from one study to another.

The results of the regression analysis showed that health facility type, patient type, antibiotics use, duration of the episode before consultation, MFP-insurance scheme, and complications with dehydration significantly affected the cost. Higher levels of health facility and inpatient care were associated with higher cost, which is in agreement with the literature [27, 28]. It has also been proven that the longer the duration that patients wait for a consultation at health facilities since the onset of the episode of diarrhea, the higher the cost. This result was consistent with Burkle et al. [27], who found in a study conducted in Bolivia that higher costs were also associated with a higher number of days that the patients waited for consultation. Children whose parents wait a long time to seek care after the onset of diarrhea may develop more severe diseases or complications, which may result in higher treatment costs. Therefore, interventions that could reduce the waiting time before initiating treatment are suggested as means to reduce cost; for example, improving access to health facilities or educating society to seek consultation as soon as patients develop diarrhea. The insurance scheme, “MFP”, which is an insurance scheme for state officials, was found to be a factor that increases cost when compared to other health insurance schemes. This is due to the payment method of this scheme, which is fee-for-service.

Although WHO discourages the use of antibiotics for diarrhea, they were prescribed to nearly 62% of patients and were significant predictors of the cost of diarrhea. Interestingly, almost all patients who visited the health center were prescribed antibiotics. Nevertheless, this treatment pattern is consistent with previous studies conducted in Rwanda [30] and South-Africa [36] where antibiotics were used at a rate of 62 and 67%, respectively.

Regarding the results of the regression analysis, savings from preventing dehydration can be applied in a cost-benefit analysis of clinical practice and disease management related to providing ORS to prevent dehydration. For instance, we demonstrated that preventing one case of dehydration can save Int$16.81. Compared to the primary treatment cost of one case in the community-based management program (cost of ORS and zinc sulfate is Int$1.48/case), our estimates show that the savings per one dehydration case prevented can be used to provide primary treatment to 11 patients (16.81/1.48 = 11.36). Assuming a coverage and an effectiveness of 100% for dehydration prevention, an ORS program can save an amount of Int$37,436,559.21 at the national level.

The community-based management program, which was recently implemented in Burundi, is an example of the ORS program recommended by WHO to manage diarrhea in developing countries [44]. The program consists of treating all cases of moderate diarrhea occurring in children aged under 5 years with oral rehydration salts and zinc by community health workers at their communities [9]. However, the the program is still limited to a few health districts. Although the evaluation of such a program was out of the scope of the present study, we demonstrated through our model that such a program could generate savings on treatment cost. Therefore, we recommend conducting a full economic evaluation of such a program as well as extension of the program to all health districts in Burundi.

In Burundi, access to health facilities seems to be adequate; the number of health centers and hospitals is estimated to be 1 health center per 10,109 inhabitants (WHO: 1 health center/10,000 inhabitants) and 1 hospital per 131,414 inhabitants (WHO: 1 hospital/150,000 inhabitants) [45]. However, access to health care is limited by the frequency of out-of-stock drugs. Stock-outs for tracer essential medicines have been observed in 45% of public health centers and 69% of public hospitals. In addition, there is unequal distribution of health professionals; most qualified health professionals are found in urban areas, while most Burundians stay in rural areas [7]. Furthermore, around 61% of Burundians are unable to pay health care fees with their usual income; they require additional sources of funding, such as working additional jobs or selling a part of their properties, or they could go into debt [46]. These factors could inhibit the promptness of treatment of diarrhea, which is a key factor to reduce cost. Therefore, in light of our regression model estimates, policymakers should improve access to health care for all Burundi inhabitants. One of the easiest ways is to improve clinical management, resulting in lower cost. Our model predicted that there would be a burden of Int$354.81 and only Int$14.49 per case of diarrhea in situations of late treatment at a hospital and early treatment at a health center, respectively. The difference (Int$340.32) between these two extreme situations shows the maximum amount that can be saved with perfect management compared to a do-nothing strategy. This illustration should be used to raise awareness of the potential loss when nothing is done as well as the potential benefit when a comprehensive intervention is implemented. In addition, this difference can be used as a key performance indicator target in the strategic management of a comprehensive health care intervention, which aims for early prevention of dehydration (ORS program), education of clinicians to use antibiotics appropriately, education of the public to seek consultation earlier, improvement of service utilization, etc. In return, the cost savings could be used in the management of the program, which would benefit the society.

While this study has several strengths, such as consistent methods and a vast range of data collected, it also has several limitations. Firstly, our study did not estimate the cost of diarrhea by etiology because this kind of data was not available at the study health facilities. Secondly, for the unit cost of routine services for outpatient visit and bed-day, we used estimates from WHO-CHOICE; although specific to the country, these estimates may not be specific to the study health facilities. Thirdly, data collected on the severity of diarrhea were limited only to the presence or absence of dehydration, even though there are many other clinical features related to severity of diarrhea. Lastly, regarding socio-economic status of the children with diarrhea that might affect the burden estimates, we just collected data on health insurance status.

## Conclusion

Diarrhea was associated with a substantial economic burden in Burundi. The cost per episode was Int$109.01, which is considerable (14.54%) compared to the people productivity (GDP per capita). Our model suggests that an ORS program to prevent dehydration, reduction of the use of antibiotics, and earlier consultation at health facilities can save a substantial amount and reduce the economic burden of diarrhea on society. This economic evidence provides important information to support the implementation of an ORS program and an effective service system for diarrhea in Burundi.

## Data Availability

All data generated or analysed during this study are included in this published article.

## Abbreviations

CMCB: Centre de Médecine Communautaire de Buyenzi
CPI: Consumer Price Index
DHS: Demographic and Health Survey
EPI: Expanded Program of Immunization
GNI: Gross National Income
GDP: Gross Domestic Product
HPRC: Prince Regent Charles Hospital
MFP: Mutuelle de la Fonction Publique
PPP: Purchasing Power Parity
ORS: Oral Rehydration Salts
SD: Standard Deviation
UNICEF: United Nations Children’s Fund
WHO: World Health Organization
WHO-CHOICE: WHO-CHOosing Interventions that are Cost-Effective
